# No Increase in Blood Pressure Assessed With the 24‐h Holter Monitoring in Patients With Episodic Migraine During Early Treatment With Anti‐CGRP Monoclonal Antibodies: A Prospective Observational Study (SAFHYPER)

**DOI:** 10.1111/ene.70351

**Published:** 2025-09-06

**Authors:** Flavia Lo Castro, Niccolò Bonini, Luigi Francesco Iannone, Alberto Boccalini, Daria Brovia, Luca Pani, Giuseppe Boriani, Simona Guerzoni

**Affiliations:** ^1^ Digital and Predictive Medicine, Pharmacology and Clinical Metabolic Toxicology‐Headache Center and Drug Abuse‐Laboratory of Clinical Pharmacology and Pharmacogenomics AOU Policlinico di Modena Modena Italy; ^2^ Cardiology Division AOU Policlinico di Modena Modena Italy; ^3^ Department of Biomedical, Metabolic, and Neural Science University of Modena and Reggio Emilia Modena Italy; ^4^ Department of Psychiatry and Behavioral Sciences University of Miami Miami Florida USA

**Keywords:** cardiovascular events, CGRP, hypertension, migraine, safety

## Abstract

**Background:**

Migraine is associated with an increased cardiovascular risk, including hypertension. Anti‐calcitonin gene related peptide (CGRP) monoclonal antibodies (mAbs) are effective preventive treatments, but concerns have been raised about their potential hypertensive effects. Herein, we assess the early changes in blood pressure (BP) during anti‐CGRP mAbs treatment in patients with migraine using 24‐h Holter monitoring.

**Methods:**

We conducted a prospective, real‐world study including 20 patients with episodic migraine (EM) during the early treatment with anti‐CGRP mAbs. Participants underwent 24‐h Holter BP monitoring before treatment and 4 weeks after the first injection. The primary outcome was the change in mean systolic BP (SBP). Secondary outcomes included changes in diastolic BP (DBP), differential BP, diurnal/nocturnal values, heart rate (HR), and dipping patterns.

**Results:**

Mean 24‐h SBP and DBP showed non‐significant differences after treatment (−2.4 mmHg and −1.8 mmHg, *p* = 0.075). No significant changes were observed in diurnal BP, but a significant reduction in nocturnal DBP was detected (−2.6 mmHg, *p* = 0.026). Consistently, the proportion of patients with a physiological dipping profile increased from 45.0% to 85.0% post‐treatment (*p* = 0.008). HR remained unchanged, and no patients had mean PAD ≥ 130/80. No adverse events were reported.

**Conclusion:**

Anti‐CGRP mAbs did not induce clinically relevant BP increases in the early treatment phase and were associated with improved nocturnal DBP and a favorable shift in dipping profile in patients with EM. These findings suggest the short‐term cardiovascular safety of anti‐CGRP mAbs, though further studies with larger cohorts and longer follow‐up are warranted.

## 1 Introduction

Migraine is a highly prevalent and disabling neurological disorder, affecting approximately 1 billion people worldwide [1]. Migraine itself is related to an elevated risk of myocardial infarction and stroke, and migraine with aura demonstrates an even stronger association with major cardiovascular disease (CVD) rates [2]. Moreover, migraine patients are at increased risk of developing hypertension [3]. These findings emphasize the importance of monitoring cardio‐ and cerebrovascular risk in migraine patients, mainly if treated using anti‐calcitonin gene‐related peptide (CGRP) pathway monoclonal antibodies (anti‐CGRP mAbs; erenumab, galcanezumab, fremanezumab, eptinezumab) [4] or gepants (rimegepant and atogepant) [5]. These drugs are currently approved by both the Food and Drug Administration (FDA) and the European Medicines Agency (EMA) for the preventive treatment of migraine and have demonstrated great efficacy and tolerability both in randomized clinical trials (RCTs) and in clinical practice. Various network meta‐analyses comparing anti‐CGRP mAbs underlined these results and showed no difference in effectiveness/efficacy or side effects [6, 7]. Recent guidelines from the International Headache Society (IHS) recommend them also as first‐line treatments [8, 9].

However, it should be considered that CGRP is widely distributed not only in the central (CNS) and peripheral nervous system (PNS), but also in the cardiovascular, respiratory, and gastrointestinal systems [10] and, in addition to playing a role in cephalic nociception, CGRP participates in several physiological processes and homeostatic responses during pathological conditions [10]. On the cardiovascular system, CGRP is a potent vasodilator having 10‐fold greater potency than prostaglandins and 10–1000‐fold greater potency than acetylcholine and substance P. Vasodilatory action is caused by the activation of CGRP‐receptors, with endothelium‐dependent mechanisms, mediated by nitric oxide, or with endothelium‐independent mechanisms, mediated by cAMP [11].

Post‐marketing case reports have raised concerns about the risk of hypertension in patients treated with anti‐CGRP mAbs. Since 2018, reports of suspected AEs related to increased blood pressure (BP) values in patients treated with erenumab have emerged [12, 13]. US Prescribing Information for erenumab was therefore updated in April 2020 to include the risk of hypertension based on post‐marketing data. Interestingly, events reported were acute and occurred most frequently (28/61, 46%) within 1 week of the first dose of erenumab [12].

However, a recent systematic review and meta‐analysis did not find an association of erenumab with significant increases in systemic BP [14]. On the other hand, another comprehensive systematic review [15] reported the impact of anti‐CGRP mAbs on BP and provided advice on potential clinical implications, suggesting that the potential risk of increased BP requires attention in patients undergoing treatment with anti‐CGRP mAbs [15]. Therefore, studies with the specific aim to assess BP in migraine patients treated with anti‐CGRP drugs are clinically needed, considering also that only four studies respected the criteria to be included in the latter systematic review [15], which excluded observational studies without a control group [16, 17]. Understanding the impact of these anti‐CGRP drugs on BP is essential for identifying at‐risk subgroups and informing personalized treatment strategies tailored to migraine characteristics, cost considerations, patient preferences, age, and comorbidities.

In the present study, we evaluated the BP changes with a rigorous 24‐h continuing monitoring (24‐Holter) impact after the first administration of subcutaneous anti‐CGRP mAbs in patients with episodic migraine (EM). We hypothesized that initiating treatment with anti‐CGRP mAbs does not result in a statistically significant change in blood pressure during early treatment.

## 2 Methods

### 2.1 Study Design

SAFHYPER is a real‐world, prospective, single‐center, investigator‐initiated, and independent study, considering all consecutive outpatients with EM treated with anti‐CGRP mAbs at the *Headache Center of Modena, Italy*.

In this study, we included all patients who provided their written consent to participate and fulfilled inclusion/exclusion criteria (see below) from October 1, 2022 to November 28, 2024. Eptinezumab and gepants were not available at the time of the study. Prophylactic therapy with anti‐CGRP mAbs was prescribed according to clinical practice, in compliance with the Italian Medicines Regulatory Agency (AIFA) reimbursement criteria.

**FIGURE 1:**
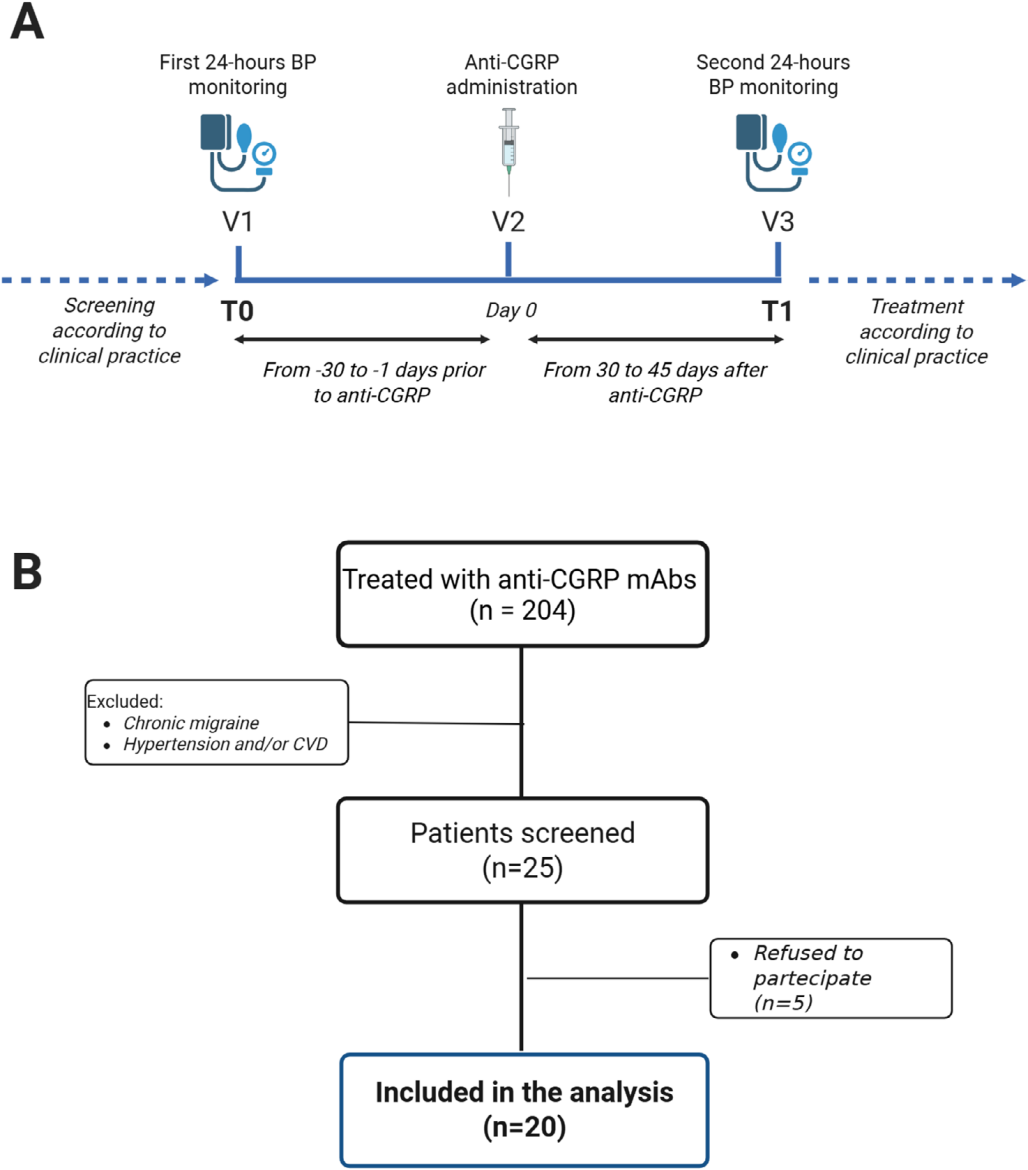
Study design (A) and flow chart of patients (B). BP, blood pressure; CGRP, calcitonin gene related peptide; CVD, cardiovascular disorders; mAbs, monoclonal antibodies.

Patients underwent a 24‐h blood pressure Holter monitoring at baseline (Visit 1 [T0]) before the start of the anti‐CGRP mAb (Visit 2, timeframe from −30 to −1 days before) and after 4 weeks of treatment (Visit 3, timeframe from 30 to 45 days after first injection). Therefore, the study assessed early treatment with anti‐CGRP mAbs. Figure 1A reported the study design.

The 24‐h BP Holter monitoring was chosen instead of the outpatient setting BP measurement, the method on which nearly all reports related to erenumab in the US and other studies are based [13, 14, 18], to eliminate biases inherent in single BP measurements and outpatient settings. It should be noted that, to diagnose arterial hypertension, according to the latest guidelines (The 2024 European Society of Cardiology [ESC] Guidelines) [19], at least three BP measurements should be taken, each1 to 22 min apart, with an additional measurement only if the readings differ by > 10 mmHg (with values ≥ 140/90 mmHg), and a minimum of two outpatient visits are required.

The local Ethics committee approved the study with the code 550/2020/OSS/AOUMO. All patients signed a written informed consent before starting the study.

### 2.2 Patient Features

Participants were enrolled regardless of the number of preventive treatments interrupted for ineffectiveness or tolerability. Ineffectiveness was defined as no meaningful improvement in migraine‐related variables after the administration of drugs for ≥ 6 weeks at the appropriate dose according to the European Headache Federation (EHF) criteria [20].

*Inclusion criteria* were: (i) patients aged 18 years or older; (ii) diagnosis of migraine without aura according to ICHD‐3 [21]; (iii) no more than 14 monthly headache days (MHDs) per month in the last 3 months; (iv) at least 8 monthly migraine days (MMDs) in the 3 months before enrollment; (v) availability of headache diaries over at least 1 month before enrollment; (vi) clinical indication for prescription of any subcutaneous anti‐CGRP mAbs (erenumab, 70 or 140 mg monthly; galcanezumab 120 mg monthly, following a loading dose of 240 mg; fremanezumab 225 mg, monthly or 675 mg, quarterly); (vii) stable treatment with other preventive treatments for migraine; (viii) presence of BP values below 139/89 mmHg during the screening visit (T0) and reported by the patients in the last 3 months.

*Exclusion criteria* were: (i) patients with any contraindications to anti‐CGRP mAbs according to the summary of product characteristics; (ii) patients with CM or migraine with aura; (iii) not stable treatment with preventive drugs for at least 3 months; (iv) pregnancy and breastfeeding; (v) patients with arterial hypertension (according to international guidelines, the 2024 European Society of Cardiology [ESC] Guidelines) [19]; (vi) patients with a history of CVD (including myocardial infarction, unstable angina, percutaneous coronary intervention, coronary artery bypass graft, or stroke), and/or at high risk for cardiovascular events; (vii) requiring adjustments/introduction of antihypertensive therapy.

Patients with CM, with aura and with controlled arterial hypertension were excluded to ensure a homogeneous study population and reduce variability in baseline cardiovascular risk. CM patients often have longer disease duration, greater use of acute medications, and are more frequently treated for other comorbidities, which could confound the assessment of early BP changes.

Patients with migraine with aura were excluded to avoid potential confounding from the distinct vascular risk profile associated with aura. Individuals with controlled arterial hypertension were also excluded to remove the influence of anti‐hypertensive therapy on BP variability and to isolate the direct cardiovascular effects of anti‐CGRP mAbs in normotensive patients.

### 2.3 Collected Variables

Clinicians diagnosed migraine and collected clinical and demographic features: concomitant and previous preventive treatments, MHDs, and the number of monthly acute medications (AMNs) at T0 (i.e., baseline). Clinical variables not related to migraine, including comorbidities, were reported according to the patient's chart and outpatient interview during clinical practice. To evaluate the overall burden of migraine, we evaluated MHDs as headache days defined as any day on which a patient recorded any type of headache. Any AEs (related or unrelated to cardiovascular system) were collected.

The 24‐h Holter BP monitoring reported: overall mean and diurnal/nocturnal systolic BP (SBP), overall mean and diurnal/nocturnal diastolic BP (DBP), overall mean and diurnal/nocturnal differential BP (automatically calculated), heart rate (HR), Dipper profile (i.e., presence of physiological dipping pattern, defined as a nocturnal SBP reduction of ≥ 10% compared to diurnal values, categorical [Y/N]).

### 2.4 Outcomes and Analysis

#### 2.4.1 Primary Outcome

Change in mean SBP as measured by 24‐h Holter BP monitoring between Visit 1 (T0) and Visit 3 (T1).

Secondary Outcomes (evaluating differences T0–T1):

- Change in mean DBP
- Change in mean overall differential BP
- Change in diurnal and nocturnal SBP
- Change in diurnal and nocturnal DBP
- Change in mean heart rate (HR)
- Change in Dipper patterns

### 2.5 Statistical Analysis

Due to the lack of data on the effect of BP on anti‐CGRP mAbs at the time of the study, as well as the use of other methods rather than 24 BP Holter monitoring, we did not perform a structured sample size calculation. The study was proposed to all outpatients that achieved inclusion and exclusion criteria and wanted to participate. Considering the small sample size, non‐parametric tests were considered more appropriate and robust, as they do not rely on the assumption of normal distribution. Thus, statistical analysis was conducted with non‐parametric tests. We reported mean [95% confidence interval or mean plus standard deviation (SD) as appropriate] or median (95% CI) for continuous variables and number (percentage) for categorical data. No imputation was planned for missing data; no missing data were present. Pre‐ and post‐treatment comparisons of continuous variables were performed using the Wilcoxon signed rank test, while the McNemar test (with continuity correction) was applied for proportions in paired samples (dipper profile). Considering the small sample size, we did not perform a separate analysis for anti‐CGRP itself and anti‐CGRP receptor mAbs.

A two‐tailed *p* < 0.05 was considered significant for all variables. Considering that variables were normally distributed, we calculated effect sizes using Cohen's *d*, with Hedges' correction to account for sample size and Phi (*φ*) for 2 × 2 chi‐squared table.

All data were analyzed using SPSS software version 29.0 (IBM Corp, SPSS Statistics, Armonk, NY, USA) and graphs designed using GraphPad Prism version 10.00 (GraphPad Software Inc., La Jolla, USA).

## 3 Results

The final study population included 20 participants with high‐frequency migraine without aura (70.0% [14/20] females, mean age 47.5 years [SD 10.1]). Figure 1B reported the flowchart of the study. Concomitant prophylactic therapy was common, with more than half of the patients (55.0% [11/20]) treated with at least one additional stable preventive drug (Table 1). Included patients did not report any known cerebro‐cardiovascular disorders or have hypertension.

**TABLE 1:** Clinical and demographic features.

	Cohort (*n* = 20)
Age years; mean (SD)	47.5 (10.1)
Female sex, % (*n*)	70.0 (14)
Concomitant preventive, % (*n*)	55.0 (11)
Clinically relevant comorbidities, % (*n*)	65.0 (13)
Psychiatric	10.0 (2)
Gastroenterological	15.0 (3)
Respiratory	15.0 (3)
Endocrinological	10.0 (2)
Sleep disorders	15.0 (3)
Anti‐CGRP mAbs, % (*n*)	
Erenumab	20.0 (4)
Galcanezumab	40.0 (8)
Fremanezumab	40.0 (8)
Migraine related variables, mean (SD)	
MHDs	11.0 (2.1)
AMNs	12.4 (4.1)

*Note:* Percentages are expressed on column total.

Abbreviations: AMD, days with at least one analgesics use per month; AMNs, number analgesics per month; MHDs, monthly headache days; SD, standard deviation.

At baseline, participants presented a mean of 11.0 ± 2.1 (SD) MHDs, and a mean of 12.4 ± 4.1 doses of AMNs. Clinical and demographic features are fully detailed in Table 1. No patients dropped out of the treatment for any reason in the first month of treatment.

### 3.1 Blood Pressure Assessment

**FIGURE 2:**
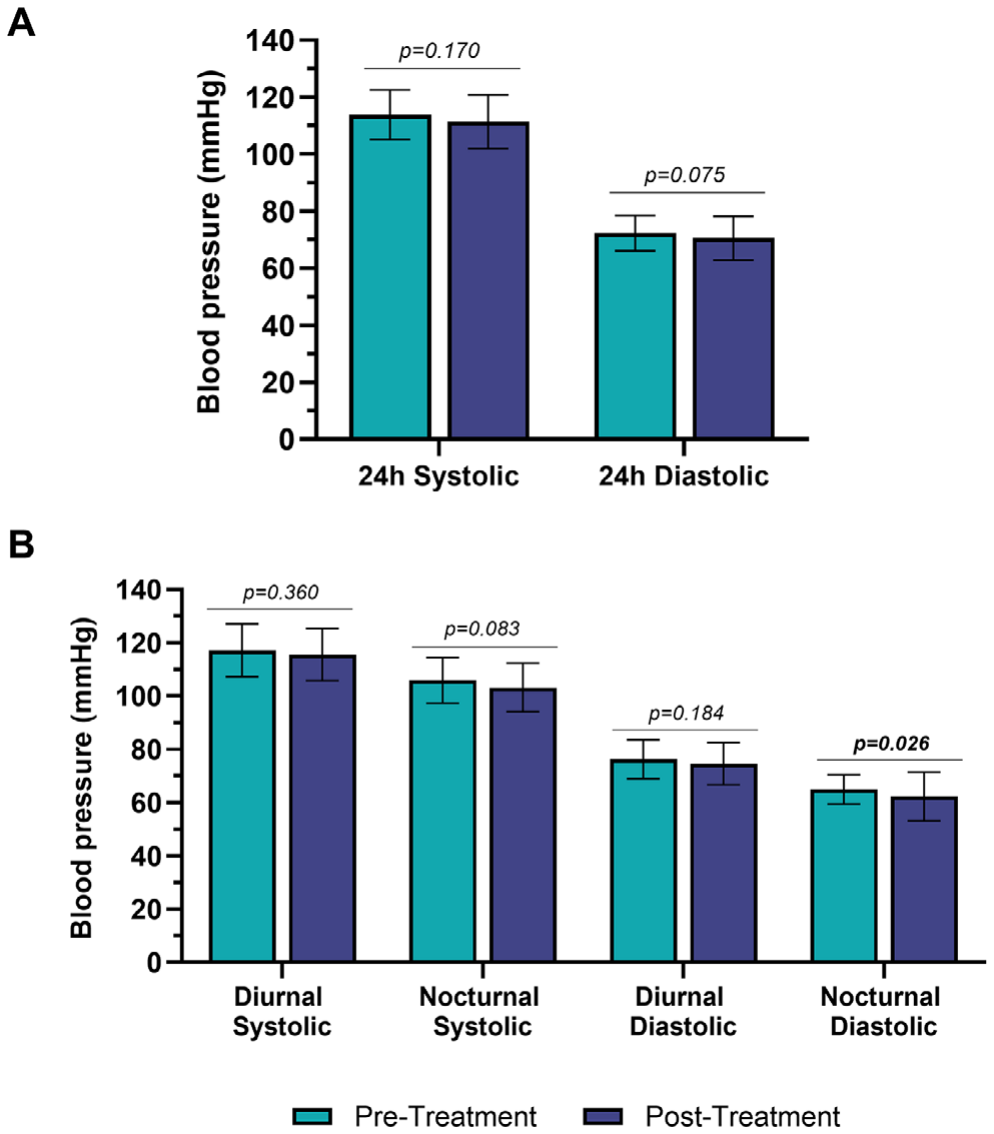
Blood monitoring in the 24 h subdivided into all day (A) and diurnal–nocturnal (B) prior and post treatment with anti‐CGRP mAbs. Values in bold are statistically significant.

BP values, assessed through 24‐h monitoring, did not significantly change following anti‐CGRP mAbs therapy. The mean 24‐h SBP decreased from 113.8 ± 8.8 mmHg at baseline to 111.4 ± 9.4 mmHg post‐treatment, with a mean reduction of 2.4 mmHg (*p* = 0.170; Cohen's *d* = 0.37). A similar reduction was observed in mean 24‐h DBP, which declined from 72.3 ± 6.2 mmHg to 70.5 ± 7.7 mmHg (*p* = 0.075; *d* = 0.43) (Table 2 and Figure 2A). Differential BP, calculated as the difference between SBP and DBP, increased from 41.8 ± 4.8 mmHg to 46.5 ± 15.8 mmHg after therapy (*p* = 0.759; *d* = −0.31), but the change was not statistically significant.

**TABLE 2:** Monitoring 24 h blood pressure values at baseline and after 4‐week therapy with anti‐CGRP mAbs.

BP (mmHg)	Pre‐treatment (mean ± SD)	Post‐treatment (mean ± SD)	*p*
24 h systolic	113.8 ± 8.8	111.4 ± 9.4	0.170
24 h diastolic	72.3 ± 6.2	70.5 ± 7.7	0.075
Differential	41.8 ± 4.8	46.5 ± 15.8	0.759
Diurnal systolic	117.2 ± 9.9	115.5 ± 9.8	0.360
Diurnal diastolic	76.3 ± 7.3	74.6 ± 7.9	0.184
Nocturnal systolic	105.9 ± 8.5	103.2 ± 9.1	0.083
Nocturnal diastolic	64.9 ± 5.5	62.3 ± 9.1	**0.026**
Heart rate (bpm)	73.7 ± 5.2	73.5 ± 8.8	0.708

*Note:* All values are reported as mean (SD). Values in bold are statistically significant.

During the diurnal phase, mean SBP changed from 117.2 ± 9.9 mmHg to 115.5 ± 9.8 mmHg (*p* = 0.360; *d* = 0.20), while mean DBP showed a modest reduction from 76.3 ± 7.3 mmHg to 74.6 ± 7.9 mmHg (*p* = 0.155; *d* = 0.33) (Table 2 and Figure 2B).

Notably, during the nocturnal period, the mean SBP decreased from 105.9 ± 8.5 to 103.2 ± 9.1 mmHg (*p* = 0.083; *d* = 0.44), and the mean DBP declined from 64.9 ± 5.5 to 62.3 ± 9.1 mmHg, reaching statistical significance (*p* = 0.026; *d* = 0.47), suggesting a mild improvement in nocturnal BP control after treatment. All results are reported in Table 2 and Figure 2A,B.

**FIGURE 3:**
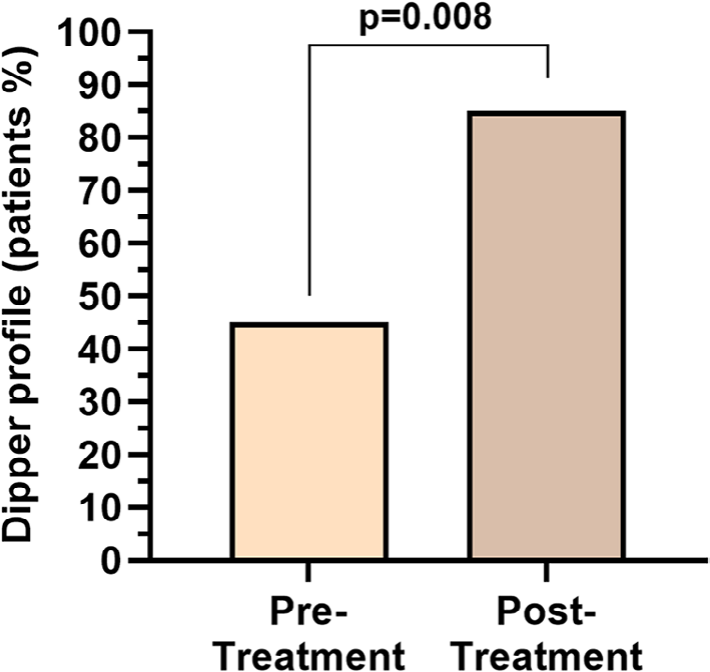
Dipper profile prior and post treatment with anti‐CGRP mAbs. Values in bold are statistically significant.

In line with that, the analysis of the dipping profile revealed a statistically significant favorable shift after treatment. At baseline, 45.0% (9/20) of patients exhibited a physiological dipping pattern, whereas post‐treatment, this proportion increased to 85.0% (17/20) (*p* = 0.008), suggesting an unexpected possible normalization of circadian BP variation following treatment (Figure 3).

Finally, no patients in the second recording had mean PAD ≥ 130/80 and the HR remained stable throughout the study period, with a mean of 73.7 ± 5.2 bpm before treatment and 73.5 ± 8.8 bpm at T1 (*p* = 0.708) (Table 2).

### 3.2 Overall Tolerability and Adverse Events Analysis

No adverse events or tolerability issues emerged during the period of the study.

## 4 Discussion

In the present study, we found no significant increase in 24‐h BP, assessed through various parameters, during the early treatment with anti‐CGRP mAbs in patients with EM. Interestingly, and unexpectedly, a statistically significant shift toward a more favorable dipping profile was observed in line with a reduction in diastolic pressure during the nocturnal period following treatment.

Several studies have evaluated BP in outpatients with mixed results; however, none have employed 24‐h monitoring to date, and none reported a beneficial shift in dipping profile and nocturnal BP.

Overall, no BP increase during treatment with anti‐CGRP mAbs, consistent with our findings, was reported in observational studies [22, 23, 24, 25, 26] and post hoc analyses [18, 27, 28], though some exceptions exist. Notably, the study by De Vries Lentsch et al. [29], which included 196 patients with migraine and a control group, found a mean increase of 5.2 mmHg in systolic BP and 3.5 mmHg in diastolic BP associated with the use of erenumab and fremanezumab, even in normotensive patients without CVD, during a 12‐month follow‐up. This study demonstrated a more pronounced and consistent effect on BP in patients treated with erenumab compared to those treated with fremanezumab [29]. Although the observed increases in mean BP were modest, cardiovascular events have a continuous relationship with BP, and even small elevations could have clinical implications [30]. Importantly, no major risk of developing hypertension was identified.

As mentioned before, the other studies [18, 22, 23, 24, 25, 26, 27, 28] did not find any increase in BP or hypertension‐related variables. It should be noted, however, that although these studies lacked a control group and did not assess potential confounding factors (such as the initiation of anti‐hypertensive therapy or baseline CVD risk) [15, 16], they included large patient populations, evaluated various aspects of the cardiovascular impact of anti‐CGRP mAbs, and one study specifically examined a large cohort of elderly patients (> 60 years) with migraine [24]. Still, as partially outlined by preclinical studies, patients with pre‐existing hypertension may be at a higher risk of developing further elevations in BP [26], suggesting a need for careful monitoring case by case. Overall, these studies provide valuable insights and are an important complement, particularly in the context of post‐marketing safety surveillance, to RCTs and post hoc analyses as well as indirect evidence from metanalysis.

A recent systematic review [14] evaluating BP changes and hypertension‐related outcomes in patients treated with erenumab found no significant association with increased systemic BP. The authors emphasized that, given the fragility of current evidence, the decision to prescribe erenumab, especially for patients with multiple comorbidities or pharmacologically controlled hypertension, should be based on a careful assessment of risks and benefits [14].

Conversely, another recent systematic review [15] highlighted the potential risk of increased BP, recommending that clinicians remain vigilant and monitor BP in migraine patients undergoing treatment with anti‐CGRP mAbs [15]. It is worth noting that these two systematic reviews applied different inclusion criteria (e.g., erenumab only vs. all anti‐CGRP mAbs, studies with control groups only vs. all available studies), thereby offering distinct perspectives on the impact of these treatments on BP.

Interestingly, the observed improvement in nocturnal DBP and the restoration of a physiological dipping profile may reflect a normalization of autonomic function in patients with migraine following anti‐CGRP mAb treatment. Although CGRP is a potent vasodilator, its blockade may paradoxically improve circadian BP regulation in migraine patients, who may display autonomic imbalance and altered baroreflex sensitivity [31]. By reducing migraine burden, anti‐CGRP mAbs may indirectly modulate sympathetic tone, restoring autonomic balance and promoting vascular homeostasis during sleep, thus normalizing nocturnal BP patterns. However, the small sample size precludes further speculation, and mechanistic studies integrating autonomic function testing with long‐term BP monitoring are needed.

From a pathophysiological perspective, extensive preclinical and clinical studies support a role for CGRP in the cardiovascular system, both under physiological conditions and in the context of cardiovascular disease, including hypertension.

In preclinical studies, administration of CGRP reduces BP values in spontaneously hypertensive rats (SHR) and in N‐nitro‐l‐arginine methyl ester‐induced hypertensive rats (L‐NAME‐HR) during pregnancy (the latter used as an animal model of preeclampsia) [32]. In male wild‐type and αCGRP knockout L‐NAME‐HRs, the hypertensive response was exacerbated in αCGRP knockout mice, indicating that endogenous αCGRP acts in a protective manner when nitric oxide production is diminished. Moreover, exogenous CGRP rescued αCGRP knockout mice from both hypertension and cardiovascular remodeling [11].

In SHRs, an age‐related decrease in the neuronal expression of CGRP was found [33], and in Dahl‐salt hypertensive rats (Dahl‐salt‐HR) a decrease in CGRP content in the dorsal ganglion was found [34]. In SHRs, CGRP receptor expression in mesenteric arteries is increased [35]; in addition, treatment with Angiotensin Converting Enzyme (ACE) inhibitors increases CGRP release [36]. These data confirm the compensatory role of CGRP in animal models of hypertension, with likely involvement of the renin‐angiotensin‐aldosterone system [37, 38].

Furthermore, CGRP‐knockout mice have significantly increased BP values compared with wild‐type mice [39, 40, 41, 42, 43], but not consistently in all studies [44, 45]. In one study, administration of a bolus of human CGRP (1.5 ng/g) resulted in a rapid decrease in mean BP in wild‐type mice and a greater decrease in BP in knockout mice [41], suggesting that the relative lack of CGRP in knockout mice increases the responsiveness of the vascular system to exogenous CGRP probably through a CGRP‐receptor‐mediated mechanism.

Even in CGRP knockout mice without basal changes in BP, there is a different response vs. wild‐type after exposition to a hypertensive insult, such as Angiotensin II, with development of hypertension, aortic hypertrophy, and endothelial dysfunction [45]. By administering CGRP, Angiotensin II‐induced hypertension can be attenuated [46, 47]. The expression of CGRP receptor, but not endogenous CGRP, appears to be increased in a mouse model of Angiotensin II‐induced hypertension [48].

Acute administration of a CGRP antagonist (CGRP_8‐37_) increases BP levels in four different animal models: the deoxycorticosterone‐salt hypertensive rats (DOC‐salt‐HR) [49], the subtotal nephrectomy‐salt hypertensive rats (SN‐salt‐HR) [50], L‐NAME‐HRs [32] and the two‐kidney one‐clip hypertensive rats (2K1C‐HR) [51].

By activating the transient receptor potential (TRP) channels (in particular, vanilloid‐1, TRPV1), capsaicin causes the release of CGRP from the capsaicin‐sensitive sensory nerve [52]. Injection of capsaicin into normotensive rats does not change their BP, although CGRP content in the dorsal nucleus decreases. When injection is accompanied by sodium overload or some alteration in renal function, there is an increase in BP [51]. These data seem to indicate that CGRP is important for BP regulation, particularly through its effect on renal function.

Studies in hypertensive patients have yielded conflicting results in relation to plasma concentrations of CGRP, which have been found to be reduced [53], increased [54], or unchanged [55]. However, these discordant results may reflect the different study populations (e.g., by severity and duration of disease or by concomitant drug treatments) or even the different laboratory methods used [56]. In patients with hypertension secondary to pheochromocytoma or aldosteronism, the concentration of CGRP is found to be increased, and it decreases concomitantly with the reduction in mean BP after adenectomy, suggesting that increased CGRP may be a compensatory mechanism in subjects with this secondary hypertension [54].

Altogether, these data suggest a role for CGRP not in the physiological control of BP (i.e., under normotensive conditions), but rather in regulation under pathological conditions. CGRP would therefore seem to have protective action in the development of hypertension. In addition, CGRP also appears to have a protective role against cardiac and renal complications of chronic hypertension [57, 58, 59]. It should be noted, however, that the use of anti‐CGRP mAbs in healthy animals did not induce significant changes in BP [60].

This is the first study to report the assessment of BP using a 24‐h method to evaluate the early impact of anti‐CGRP treatment and show a favorable reduction in nocturnal diastolic BP in line also with a more favorable dipping profile. The strengths of our study include its prospective design, the use of standardized 24‐h Holter BP monitoring, which provides several diurnal and nocturnal variables, and the thorough assessment of CDV risks, including all patients to better delineate anti‐CGRP's potential impact.

However, our study has several limitations, including the absence of a predefined sample size, the lack of a control group, the exclusion of patients with a concomitant stable hypertensive treatment, the single center recruitment, and a design that cannot fully account for confounding variables. The single‐group, pre‐post study design can not also separate the direct pharmacological effect of the anti‐CGRP mAb from the physiological consequences of successful migraine treatment.

The exclusion of patients with CM, migraine with aura, and controlled arterial hypertension was due to create a more homogeneous study population and reduce potential confounding factors. Chronic migraine and migraine with aura could be associated with distinct clinical and vascular risk profiles and different (and higher) use of acute medications (that could impact BP), while anti‐hypertensive therapy in patients with controlled hypertension could alter BP dynamics independently of anti‐CGRP treatment. However, these exclusions limit the generalizability of our findings to the broader migraine population. Future studies should include these patient groups to enable subgroup analyses.

Finally, the possibility that a longer treatment with anti‐CGRP may be needed to observe any differences in BP should be considered due to the potential additive effect of long‐term inhibition of the CGRP pathway [10, 61].

While the absence of a control group is a key limitation, future research could address this gap. For instance, patients initiating anti‐CGRP mAbs could be compared with matched migraine patients receiving alternative preventive therapies or no treatment/placebo, with both groups undergoing baseline and follow‐up 24‐h blood pressure monitoring. Such a design would allow differentiation between the direct pharmacological effects of anti‐CGRP mAbs and changes related to migraine improvement or other external factors, thereby strengthening causal inference and the assessment of cardiovascular safety. Incorporating long‐term follow‐up with repeated 24‐h monitoring would also be essential to capture delayed or cumulative BP effects that may not emerge in the early treatment phase.

## 5 Conclusions

Our study is in line with the overall safety and tolerability of anti‐CGRP mAbs with respect to blood pressure modification during early treatment, as monitored through 24‐h continuous assessment. These results may reinforce the cardiovascular safety of anti‐CGRP mAbs, an important consideration given that migraine is recognized as a significant cardiovascular risk factor in women. Nevertheless, a careful evaluation, before starting an anti‐CGRP drug in patients with pre‐existing hypertension, should be carefully designed to delineate the benefit/harm risk in this population. Further studies with longer follow‐up periods and similarly thorough BP monitoring, involving patients with CM and/or aura and treated with both anti‐CGRP mAbs and gepants, are highly anticipated.

## Author Contributions

F.L.C., N.B., and L.F.I. had full access to all of the data in the study and take responsibility for the integrity of the data and the accuracy of the data analysis. F.L.C. designed the study and drafted the manuscript. L.F.I. performed statistical analysis and drafted the manuscript. All authors critically reviewed the manuscript, agreed to be fully accountable for ensuring the integrity and accuracy of the work, and read and approved the final manuscript.

## Ethics Statement

The local Ethics committee approved the study with the code 550/2020/OSS/AOUMO.

## Conflicts of Interest

L.F.I. received financial support, consulting fees for participation in advisory boards, and support for attending meetings from: Teva, Eli Lilly, Lundbeck, Organon, Pfizer, and AbbVie; he is Associate Editor for Frontiers in Neurology Headache and Neurogenic Pain section. S.G. has received fees and honoraria for advisory boards, speaker panels, or clinical investigation studies from Novartis, Teva, Eli‐Lilly, Pfizer, Lundbeck, Angelini, AbbVie, Orion, and Organon. G.B. has received speaker's fees from Boston, Bayer, Daiichi‐Sankyo, Sanofi, and Janssen outside the submitted work. Other authors have no relevant financial or non‐financial interests to disclose.

## Data Availability

The data that support the findings of this study are available from the corresponding author upon reasonable request.
